# *SYK*-targeted dendritic cell-mediated cytotoxic T lymphocytes enhance the effect of immunotherapy on retinoblastoma

**DOI:** 10.1007/s00432-018-2584-x

**Published:** 2018-01-25

**Authors:** Xuemei Chen, Patricia Elena Kunda, Jianwei Lin, Meiling Zhou, Jinghan Huang, Huqin Zhang, Tao Liu

**Affiliations:** 1 The Key Laboratory of Biomedical Information Engineering of Ministry of Education, School of Life Science and Technology, Xi’an Jiaotong University, Xi’an, 710049 China; 2 Centro Investigación Medicina Traslacional “Severo Amuchástegui” (CIMETSA), Instituto Universitario Ciencias Biomédicas Córdoba (IUCBC), Córdoba, Argentina; 3 Shenzhen Key Laboratory for Anti-Ageing and Regenerative Medicine, Health Science Center, Shenzhen University, 3688 Nanhai Avenue, Shenzhen, 518060 Guangdong China; 4 Department of Biotherapy, Shenzhen Luohu People’s Hospital, No. 47 Youyi Road, Shenzhen, 518001 Guangdong China

**Keywords:** Retinoblastoma, Spleen tyrosine kinase, Dendritic cells, Cytotoxic T lymphocytes, Autologous adoptive immunotherapy

## Abstract

**Purpose:**

Retinoblastoma (RB) is the most common primary intraocular tumor in children. Chemotherapy is currently the main method of RB treatment. Unfortunately, RB often becomes chemoresistant and turns lethal. Here, we used in vitro cell immunotherapy to explore whether adoptive immunotherapy could be used as a potential treatment for RB. We focused on spleen tyrosine kinase (SYK), which is significantly upregulated in RB cells and serves as a marker for RB cells.

**Methods:**

Using lentiviruses, we genetically modified dendritic cells (DCs) to express and present the SYK peptide antigen to cytotoxic T lymphocytes (CTLs) in vitro. We used SYK-negative cell lines (MDA-MB-231, MCF-10A, and hTERT-RPE1) and SYK-positive cell lines (MCF-7 and RB-Y79) to evaluate the specificity and cytotoxicity of DC presented CTLs using FACS, live-cell imaging, and RNA interference.

**Results:**

The cytotoxicity of CTLs induced by SYK-overexpressing DCs (SYK-DC–CTLs) was enhanced more than three times in SYK-positive cell lines compared with SYK-negative cell lines. DCs primed with SYK could drive CTL cytotoxicity against SYK-positive cell lines but not against SYK-negative cell lines. Moreover, *SYK*-silenced RB-Y79 cells successfully evaded the cytotoxic attack from SYK-DC–CTLs. However, SYK-DC–CTLs could target SYK overexpressed hTERT-RPE1 cells, suggesting that SYK is a specific antigen for RB. Furthermore, SYK-DC–CTL exhibited specific cytotoxicity against carboplatin-resistant RB-Y79 cells in vitro.

**Conclusions:**

Our data showed that SYK could be a potential immunotherapy target mediated by DCs. We propose SYK as a candidate target for treatment of chemoresistant RB.

**Electronic supplementary material:**

The online version of this article (10.1007/s00432-018-2584-x) contains supplementary material, which is available to authorized users.

## Introduction

Retinoblastoma (RB) is the most common primary intraocular tumor in children and is initiated by the bi-allelic loss of *RB* gene function (Sachdeva and O’Brien 2012). RB is highly aggressive and leads to intraorbital, intracranial, and even systemic metastasis (Shields et al. 2013). Despite the advances made in radiation and chemotherapy along with surgical resection for the treatment of RB, the prognosis for patients with advanced RB remains poor.

Chemotherapy is currently used as first-line treatment for RB. Although this strategy could save patient lives, the treatment still has several limitations. First, eyeball enucleation and radiotherapy lead to blindness, disablement, and an inferior quality of life. Second, chemotherapy causes serious side effects such as myelosuppression, neutropenia, infection, anemia, and hearing loss. Finally, long-term chemotherapy leads to multidrug resistance, which increases the chances of recurrence and metastases (Shields et al. 2003). These disadvantages indicate the need for new and effective therapeutic approaches for RB without limiting side effects.

The spleen tyrosine kinase (*SYK*) was recently identified as one of the most significantly upregulated kinase genes in RB (Zhang et al. 2012). SYK is involved in B-cell receptor complex signaling in various inflammatory cells and has been implicated in hematopoietic cell malignancies (Chen et al. 2008; Feldman et al. 2008; Hahn et al. 2009; Young et al. 2009). There are two SYK isoforms in tumor cells: the full-length SYK (SYK-L) and the alternatively spliced SYK transcript (SYK-S). SYK-L can enter the nucleus and suppress cancer cell invasiveness, whereas SYK-S is restricted to the cytoplasm and could promote tumor progression (Wang et al. 2003). *SYK* is also a proto-oncogene involved in RB cell survival. However, *SYK* is not expressed in either retinal progenitor cells or neurons and has no known function in the developing visual system. These observations suggest that this gene might drive RB tumorigenesis (Zhang et al. 2012). Thus, *SYK* could be a suitable candidate for RB therapy.

Adoptive immunotherapy has been shown to possess great potential as an adjuvant treatment to control cancer (Sachdeva and O’Brien 2012). One of the key players in mediating the immune response are the dendritic cells (DCs), as they prime näive helper and cytotoxic T lymphocytes (CTLs) (Ahmed and Bae 2014). DCs can capture, process, and present antigens to T cells and trigger a specific anti-tumor autoimmune response (Banchereau and Steinman 1998). However, malignancies can inactivate DCs by expressing immune inhibitory molecules and/or by secreting immunosuppressive cytokines, thus leading to ineffective antigen presentation to DCs. Ultimately, this inactivation of DCs allows tumor cells to evade anti-tumor immunological responses (Ahmed and Bae 2014; Nestle 2000). To overcome this limitation, in vitro-generated functional DCs have been intensively researched over the past decade (Palucka and Banchereau 2012). These DCs can be loaded with antigens, a procedure that increases DC specificity and enhances the targeting and killing of cancer cells (Liu et al. 2013; Wang et al. 2013).

In this study, we genetically modified DCs, so they can persistently present antigenic epitopes on their surface, thus more strongly and specifically stimulating an anti-tumor immune response (Alexandrescu et al. 2010). We used lentiviral vectors that have been modified to be safely used in gene therapy in vivo (Wang et al. 2010). Using this strategy, we expressed SYK to prime T lymphocytes. Importantly, the DCs transfected with lentiviral vectors can activate specific anti-tumor immune responses (Ahmed Ali et al. 2014; Cui et al. 2012; Lopes et al. 2006; Wang et al. 2010; Xiao et al. 2012). We aimed to investigate whether: (1) *SYK* can be used as a specific target for RB; (2) cell immunotherapy is an effective and safe approach for RB treatment; and (3) presenting DCs with lentivirus could promote T-lymphocyte maturation and increase specific cytotoxicity against RB-Y79 cells in vitro.

## Materials and methods

### Cell lines

Human retinoblastoma cells (RB-Y79, ATCC, USA) and human retinal pigment epithelium cells (hTERT-RPE1, JENNIO Biological Technology, China) were maintained in RPMI 1640 (Thermo Fisher Scientific, USA) supplemented with 10% fetal bovine serum (FBS, Thermo Fisher Scientific, Australia). Carboplatin-resistant RB-Y79 cells (RB-Y79-R) were cultured in RPMI 1640 containing 10% FBS and 40 µg/ml carboplatin. MDA-MB-231, MCF-10A, and MCF-7 breast cancer cell lines were purchased from Shanghai Zhong Qiao Xin Zhou Biotechnology Company. Human embryonic kidney 293FT cells (Thermo Fisher Scientific, USA) were cultured in high glucose DMEM (Thermo Fisher Scientific, USA) containing 10% FBS, 0.1 mM MEM Non-essential Amino Acids (Thermo Fisher Scientific, USA), 2 mM l-glutamine (Thermo Fisher Scientific, USA), 1 mM sodium pyruvate (Thermo Fisher Scientific, USA), and 500 µg/ml geneticin (G418, Thermo Fisher Scientific, USA).

### Production of lentivirus

*SYK* cDNA (NM003177) was obtained from OriGene Technologies. The ViraPower™ HiPerform™ Lentiviral TOPO Expression Kit (Thermo Fisher Scientific, USA) and 293FT cells were used to produce SYK lentivirus, GFP lentivirus, and SYK–GFP lentivirus according to the manufacturer’s instructions. The SYK lentivirus, GFP lentivirus, or SYK–GFP lentivirus were harvested and filtered through a 0.45 µm membrane, aliquoted, and stored at − 80 °C until use.

### Generation of different types of DC–CTLs

DCs were induced by a previously described method (Wang et al. 2013). Briefly, PBMCs were isolated from blood with Ficoll–Hypaque (Morecell Biomedical Co. Ltd, Shenzhen, China). The adherent PBMCs were cultured in serum-free medium for immune cells (MCM-001, Morecell Biomedical Co. Ltd, Shenzhen, China) supplemented with 100 ng/ml GM-CSF, 30 ng/ml IL-4, and 5% autologous plasma for induction of DCs. The medium was changed every 3 days. TNF-α (10 ng/ml) was added to DCs on the 6th day. The non-adherent PBMCs were cultured in serum-free medium for immune cells containing 1000 U/ml IL-2, 1000 IU/ml IFN-γ, 200 ng/ml CD3 monoclonal antibody, and 5% autologous plasma for generating CTLs.

On the 3rd day of DCs culture, the immature DCs were infected by SYK lentivirus or GFP lentivirus at MOI 30 using 10 µg/ml hexadimethrine bromide (polybrene, Sigma-Aldrich, St. Louis, USA) (Seitz et al. 1998). 24 h later, the medium was replaced by fresh medium. On the 8th day, the mature SYK-DCs (DCs infected with SYK lentivirus) or GFP-DCs (DCs infected with GFP lentivirus) were harvested.

To generate RB-Y79 complete antigen-pulsed DCs (Ag-DCs), RB-Y79 lysates were generated by three rapid freeze–thaw cycles. Briefly, RB-Y79 cells were resuspended in PBS at the density of 1 × 10^7^ cells/ml. The RB-Y79 cells in suspension were frozen in liquid nitrogen and disrupted by three freeze–thaw cycles. The cell lysis was centrifugated for 10 min at 600*g*. The supernatant (RB-Y79 complete antigen) was collected and stored at − 80 °C. Then, the RB-Y79 complete antigen was added to immature DCs on the 3rd day. 24 h later, the medium was replaced by fresh medium. On the 8th day, the mature Ag-DCs were then harvested.

The mature wild-type DCs (WT-DCs), SYK-DCs, GFP-DCs, and Ag-DCs were co-cultured with CTLs at a ratio of 1:10 (DC: T) for another 6–8 days to generate WT-DC–CTLs, SYK-DC–CTLs, GFP-DC–CTLs, and Ag-DC–CTLs separately.

### Flow cytometry

DCs were identified with a panel of antibodies: CD80-PE, CD83-APC, and CD86-FITC (BD Bioscience). T lymphocytes were phenotyped with antibodies against CD3, CD8, CD38, HLA–DR, and T-cell receptor (TCR)-αβ (BD Biosciences, New York, USA). A mouse IgG (PE/FITC/APC) was used as a negative control in all assays. Flow cytometric analysis was performed on a FACSCalibur Flow Cytometer (BD Bioscience, New York, USA) following our previously described protocol (Liu et al. 2013).

For cytotoxicity analysis, target cells (1 × 10^5^ cells; MDA-MB-231, MCF-10A, MCF-7, hTERT-RPE1, or RB-Y79) were stained with 2 µM 5-carboxyfluorescein diacetate succinimidyl ester (CFSE). The CFSE-labeled target cells were washed and co-cultured with 2 × 10^6^ CTLs. At 24 h later, the cells were washed twice and stained with 1 µg/ml propidium iodide (PI). The Annexin V Apoptosis Detection Kit (BD Bioscience, New York, USA) was used to evaluate apoptosis according to the manufacturer’s instructions. The dual-color flow cytometry analysis was performed with FACSCalibur Flow Cytometer. To calculate the cytotoxicity in above experiments, we have already deduced the spontaneous mortality of both control and study group from the flow cytometry cytotoxicity read out in this work.

### RNA interference of RB-Y79 cells

RB-Y79 cells were transfected with *SYK*-homo-301 and *SYK*-homo-924 siRNAs (Genepharm, Shanghai, China) at 100 nM using Lipofectamine® 3000 (Thermo Fisher Scientific, USA) according to the manufacturer’s instructions. Scrambled siRNA was used as a negative control. Cells were cultured for 72 h and then used in the experiments. To avoid off-target effects, we used two pairs of siRNAs to carry out the RNA interference: *SYK*-homo-924, 5′-GUCGAGCAUUAUUCUUAUATT-3′ and 5′-UAUAAGAAUAAUGCUCGACTT-3′, as well as SYK-homo-301, 5′-GCAUGAGUGAUGGGCUUUATT-3′ and 5′-UAAAGCCCAUCACUCAUGCTT-3′. The sequences of control siRNAs (scrambled negative control) were 5′-UUCUCCGAACGUGUCACGUTT-3′ and 5′-ACGUGACACGUUCGGAGAATT-3′.

### SYK overexpression in hTERT-RPE1 cells

The hTERT-RPE1 cells were infected by SYK–GFP lentivirus at MOI 5 with the addition of 10 µg/ml polybrene. After 24 h, 1 µg/ml Puromycin (Thermo Fisher Scientific, USA) was added to the media to select the stable SYK–GFP–RPE1 cell line.

### Immunofluorescence

Immunostaining was performed followed with our previously described method (Liu et al. 2013) but not the permeabilization step. The antibodies included SYK antibody (1:400, Cell-Signaling Technology, Danvers, USA), actin antibody (1:400, Sigma, St. Louis, USA), FITC-labeled goat anti-rabbit IgG, Alexa Fluor 555-labeled donkey anti-rabbit IgG, Alexa Fluor 350-labeled goat anti-mouse IgG (1:400, Beyotime Institute of Biotechnology, China), and TRITC-conjugated Phalloidin (1:1000, Sigma, St. Louis, USA).

### Live-cell video microscopy

We previously established a stable fluorescent RB-Y79 cell line expressing the LifeAct-GFP protein (RB-Y79–GFP) (Wang et al. 2013). For live-cell video experiments, RB-Y79–GFP cells (5 × 10^3^ cells) were plated on glass-bottom dishes (MatTek, MA, USA) and kept at 37 °C in a humidified incubator for 2 h to adhere. Then, SYK-DC–CTLs were added at a ratio of 10:1. After co-culture for 12 h, cells were imaged every 5 min for 6–8 h at 37 °C using a Leica microscope. Images were then processed offline with the Image J software K1.45.

### Quantitative real-time polymerase chain reaction (qRT-PCR)

*SYK* and *GFP* mRNA levels were determined by qRT-PCR. Total RNA was obtained from 1 × 10^6^ cells using the Aurum™ Total RNA Mini Kit (Bio-Rad, California, USA). Then, cDNA was synthesized by reverse transcription from 1 µg total RNA using the iScript cDNA Synthesis Kit (Bio-Rad, California, USA). The iQ SYBR Green SuperMix Kit (Bio-Rad, California, USA) was used for real-time PCR reaction according to the manufacturer’s instructions. Glyceraldehyde-3-phosphate dehydrogenase (*GAPDH*) or *18S rRNA* was used as internal control. The nucleotide sequences of the qRT-PCR primers are listed in Table 1.

**Table 1:** Nucleotide sequences of the primers used for qRT-PCR analyses

Primer	Sequence
SYK forward	5′-TCAGCGGGTGGAATAATCTC-3′
SYK reverse	5′-TGCAAGTTCTGGCTCATACG-3′
GFP forward	5′-CACATGAAGCAGCACGACTT-3′
GFP reverse	5′-GATGCGATTCACCAGGGTAT-3′
GAPDH forward	5′-CGCATCTTCTTGTGCAGT-3′
GAPDH reverse	5′-AATGAAGGGGTCGTTGATGG-3′
18S rRNA forward	5′-AACTTTCGATGGTAGTCGCCG-3′
18S rRNA reverse	5′-CCTTGGATGTGGTAGCCGTTT-3′

### Statistical analysis

All data are presented as mean ± SEM. Because of the small sample size, the data failed to pass the D’Agostino–Pearson’s normality test. Thus, the significance of the difference between groups was evaluated with Mann–Whitney *U* test using the SPSS Statistics 19.0 software. A value of *p* < 0.05 was considered statistically significant.

## Results

### SYK lentivirus enhances DC maturation and T-lymphocyte activation in vitro

**Fig. 1:**
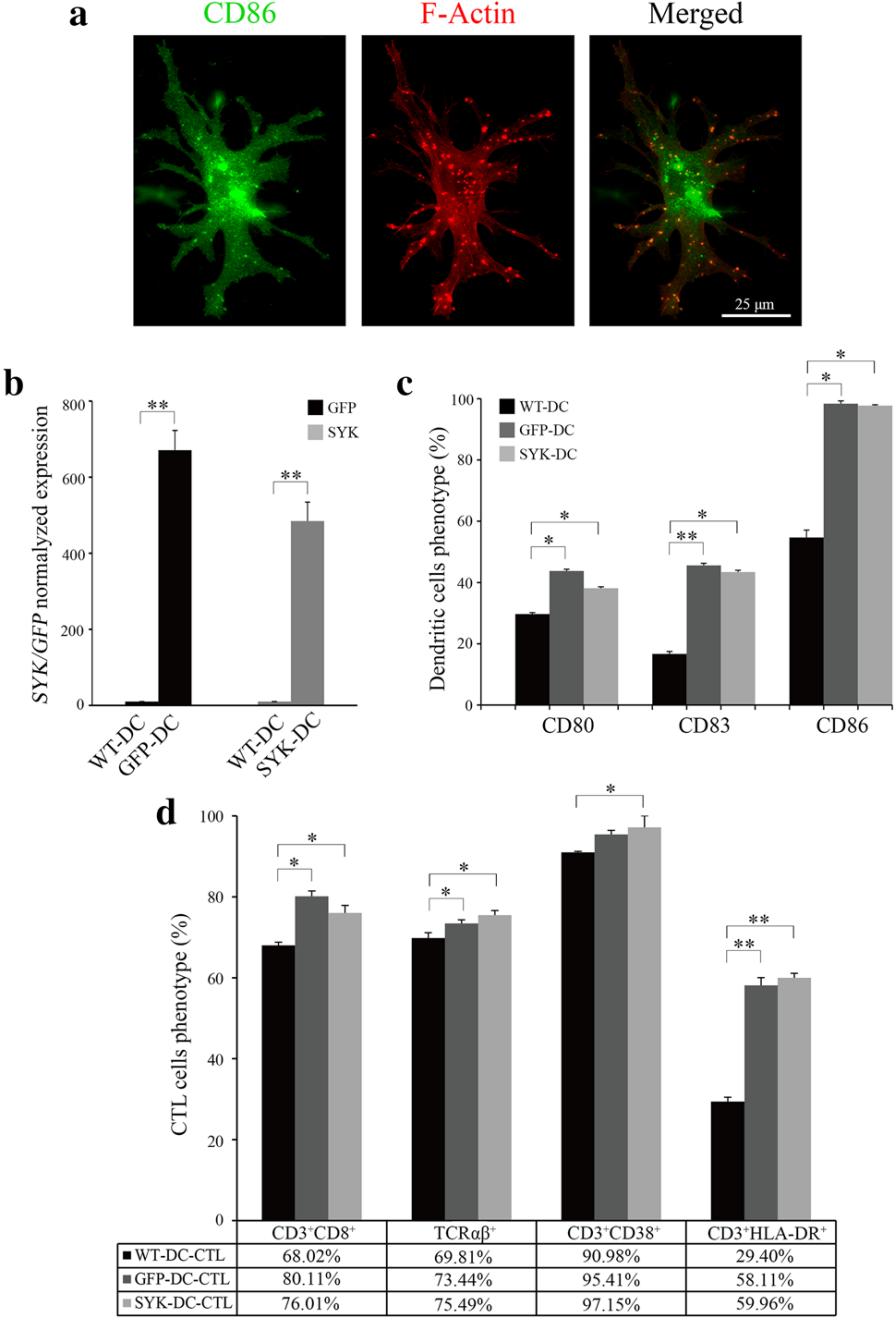
SYK lentivirus enhances DC maturation and T-lymphocyte activation in vitro. **a** CD86 marker (green signal) was spread all over the surface of DCs (left image). Numerous dendritic actin protrusions extended from the cell body (red, middle and right images). Scale bar equals 25 µm. **b** Normalized expression level of *GFP* in GFP-DCs and *SYK* in SYK-DCs. *GAPDH* was used as the internal control gene. **c** Quantification of CD80, CD83 and CD86 expression in WT-DCs, GFP-DCs, and SYK-DCs. **d** Proportions of CD3^+^CD8^+^, TCRαβ^+^, CD3^+^CD38^+^, and CD3^+^HLA–DR^+^ in WT-DC–CTLs, GFP-DC–CTLs, and SYK-DC–CTLs. Data were mean ± SEM from three independent experiments. *p < 0.05, **p < 0.01

To develop functional antigen-loaded DCs, we first generated CTLs from peripheral blood mononuclear cells (PBMCs). CTLs are commonly CD8^+^ and as such are able to recognize the complex comprising the major histocompatibility complex class I (MHC-I) molecule and the tumor antigen peptide on tumor cells, thus stimulating an effective and specific anti-tumor immune response (Ahmed and Bae 2014; Itoh and Shichijo 2005). We developed lentivirus expressing the *SYK* gene (Liu et al. 2013) and used the lentivirus to infect DCs and generate SYK-DCs. GFP lentivirus was used to establish GFP-DCs as a control. Representative images of cell morphology are shown in Fig. 1. As shown in Fig. 1a, numerous dendritic actin protrusions extended from the cell body (middle and right images), which is the typical morphology of a mature DC. The numerous actin dots (Fig. 1a, middle and right images) indicate the podosoma of DCs (Vignjevic and Montagnac 2008; Yamakita et al. 2011). They were well known as their strong migration ability and antigen assimilating (Baranov et al. 2014). Mature DCs were identified by CD86 immunofluorescent staining, characterized by a green dotted appearance (Fig. 1a, left image) spreading all over the cell surface (Ohkuma et al. 2015).

To confirm lentivirus-mediated expression, we examined GFP-DCs and SYK-DCs by qRT-PCR analysis at 7 days after infection. *SYK* expression in non-infected wild-type DCs (WT-DCs) was negligible. In contrast, SYK-DCs showed over 400 times higher expression of *SYK* compared with WT-DCs (Fig. 1b). The *GFP* and *SYK* gene expression real-time amplification curves of WT-DCs, GFP-DCs, and SYK–DCs cells are shown in Supplemental Fig. 1a.

CD80, CD83, and CD86 are commonly used as markers of mature DCs. We evaluated the expression of these markers in DCs and found significantly different between the GFP-DC and SYK–DC groups compared with the control WT-DC group (Fig. 1c). The proportions of CD80^+^, CD83^+^, and CD86^+^ cells were 29.67 ± 0.50, 16.70 ± 0.80, and 54.70 ± 2.40% in the WT-DC group, 43.78 ± 0.56, 45.55 ± 0.69, and 98.34 ± 0.91% in the GFP-DC group, and 38.11 ± 0.50, 43.43 ± 0.54, and 97.73 ± 0.25% in the SYK-DC group, respectively. These data mean overexpression of SYK results in a higher degree state of DC maturation.

We next co-cultured autologous T cells with mature WT-DCs, GFP-DCs and SYK-DCs for 7 days separately to obtain WT-DC–CTLs, GFP-DC–CTLs, and SYK-DC–CTLs. T-cell markers (CD3, CD8, and TCRαβ) are used as activation markers, while these are molecules that been expressed on all T cells including the cells with resting or naïve phenotype. CD38 and HLA-DR are commonly used to identify activated T cells (Loewendorf et al. 2014; Tarbox et al. 2014). Testing the levels of these markers is important, because they are crucial for antigen recognition and specific cytotoxicity. Thus, we examined the proportions of CD3^+^CD8^+^, TCRαβ^+^, CD3^+^CD38^+^, and CD3^+^HLA-DR^+^ populations using flow cytometry to demonstrate the activity of different T-cell groups. Higher proportion of CD3 + CD8+, TCRαβ+, CD3 + CD38 +, and CD3 + HLA-DR + cells were detected, which proportion of CD3^+^CD8^+^, TCRαβ^+^, CD3^+^CD38^+^, and CD3^+^HLA-DR^+^ populations were 68.02 ± 1.56, 69.81 ± 0.65, 90.98 ± 1.34, and 29.40 ± 1.56% in the WT-DC–CTL group, 80.11 ± 0.90, 73.44 ± 0.74, 95.41 ± 0.86, and 58.11 ± 1.27% in the GFP-DC–CTL group, and 76.01 ± 1.47, 75.49 ± 0.62, 97.15 ± 1.10, and 59.96 ± 1.12% in the SYK-DC–CTL group, respectively, as shown in Fig. 1d. This phenotype prove that SYK-DC could active T cells better than wild-type DC.

Taken together, these results demonstrated that the *SYK* gene was successfully expressed in DCs by lentivirus infection and that SYK-DCs generated in vitro could stimulate naïve T cells to become active and express higher levels of CD3^+^CD8^+^, TCRαβ^+^, CD3^+^CD38^+^, and CD3^+^HLA-DR^+^ compared with controls. The flow cytometry scatter plots are shown in Supplemental Fig. 2.

### SYK-DC–CTLs show specific cytotoxicity against SYK-positive cells but not SYK-negative cells in vitro

**Fig. 2:**
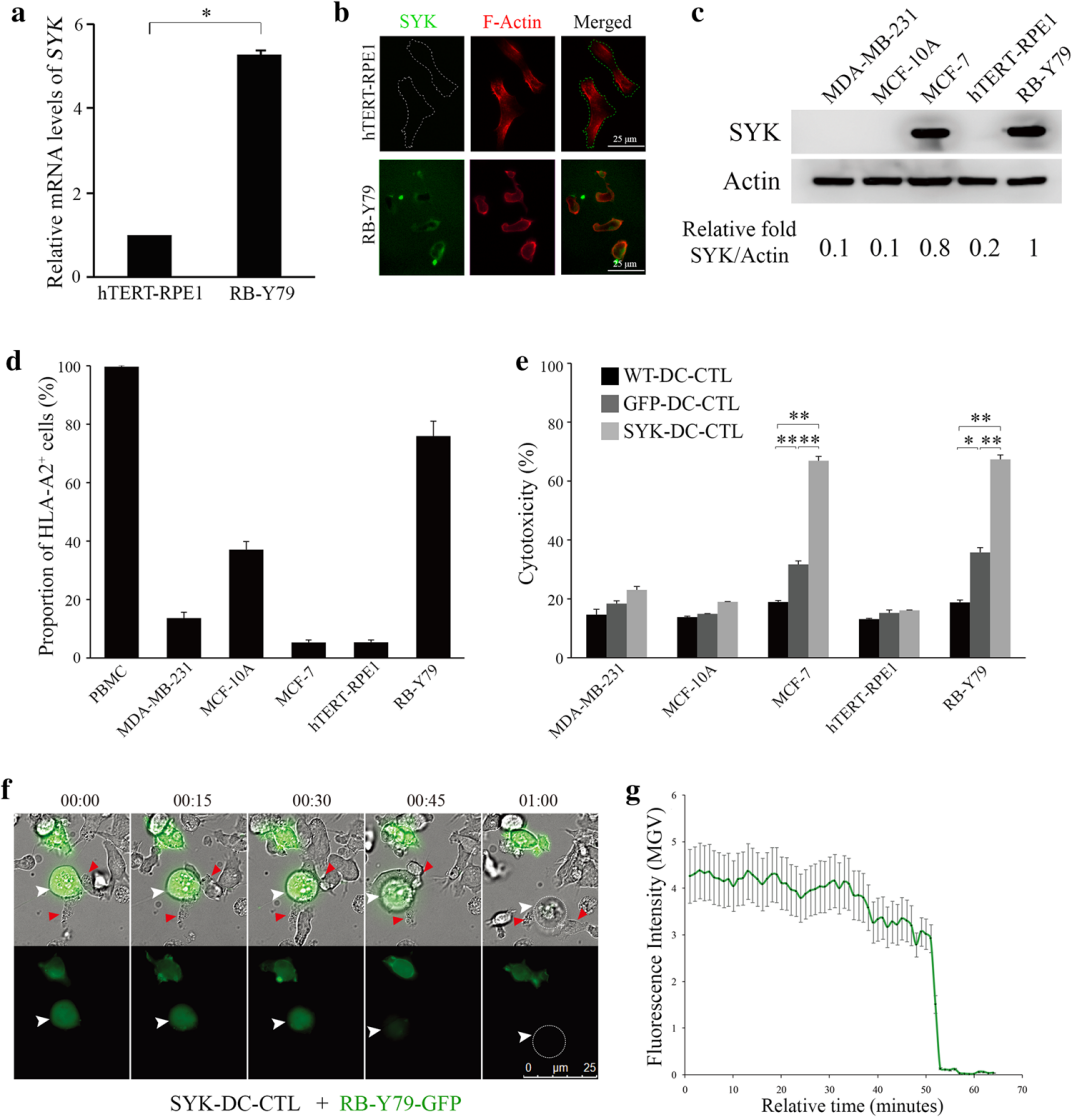
SYK-DC–CTLs show specific cytotoxicity against SYK-positive cells but not SYK-negative cells in vitro. **a** Quantification of qRT-PCR results of *SYK* expression in RB-Y79 cells and hTERT-RPE1 cells. *18S rRNA* was used as the internal control gene. *p < 0.05. **b** Immunofluorescence results showed SYK (green signal) expression on the surface of RB-Y79 cells (bottom panel). No SYK expression was detected on the surface of hTERT-RPE1 cells (top panel). Scale bar equals 25 µm. **c** Western blot analysis of SYK protein expression levels in MDA-MB-231, MCF-10A, MCF-7, hTERT-RPE1, and RB-Y79 cells. Actin was used as a loading control. **d** Flow cytometry analysis of HLA-A2 in PBMC, MDA-MB-231, MCF-10A, MCF-7, hTERT-RPE1, and RB-Y79 cells. **e** Flow cytometry analysis of apoptosis in MDA-MB-231, MCF-10A, MCF-7, hTERT-RPE1, and RB-Y79 cells co-cultured with WT-DC–CTL, GFP-DC–CTL, and SYK-DC–CTL cells. **f** Time-lapse video of SYK-DC–CTL cells (red arrow) co-cultured with an RB-Y79-GFP cell (green, white arrow). Scale bar equals 25 µm. **g** Mean gray value (MGV) of fluorescence intensity of RB-Y79-GFP cells in the time-lapse video above. Data were given as mean ± SEM from three independent experiments. *p < 0.05, **p < 0.01

To examine the effect of SYK in CTL-specific cytotoxicity against RB cells, we first evaluated basal SYK expression levels in human RB RB-Y79 and human retinal pigment epithelium cells hTERT-RPE1 cell lines using qRT-PCR and an immunofluorescence assay. Figure 2a shows that the normalized expression level of *SYK* in RB-Y79 cells was five times higher than that of hTERT-RPE1 cells (*p* < 0.05). The real-time amplification curves are shown in Supplemental Fig. 1b. Immunofluorescence results further revealed that SYK was expressed on the surface of RB-Y79 cells, but it was undetectable on the surface of hTERT-RPE1 cells (Fig. 2b). Although the immunofluorescent picture in Fig. 2b showing SYK on the cell surface of RB-Y79 displays a high background, we provided a “no primary antibody" control to show the specific staining with the antibody shown as Supplemental Fig. 3a.

Using different cell lines, we compared the consistency of SYK expression levels which is important for the specificity of cytotoxicity. As RB-Y79 is the only available malignant RB cell line, we also tested the universal property of SYK-DC–CTL. SYK high expression cell lines MCF-7 original from breast cancer was checked by western blotting using SYK-negative cell lines MDA-MB-231 and MCF-10A as controls (George et al. 2005; Mahabeleshwar and Kundu 2003). As shown in Fig. 2c, MCF-7 and RB-Y79 cells clearly showed high expression of SYK protein.

As the DC–CTL response is largely dependent on HLA types (Sui et al. 2015), we checked the histocompatibility of DC–CTL (original PBMC substitute) with target cell lines. As shown in Fig. 2d, the proportion of HLA-A2^+^ cells in PBMCs, MDA-MB-231, MCF-10A, MCF-7, hTERT-RPE1, and RB-Y79 cells was 99.66 ± 0.44, 13.64 ± 2.02, 37.07 ± 2.78, 5.29 ± 0.89, 5.36 ± 0.79, and 75.90 ± 5.11%, respectively. The flow cytometry scatter plots are shown in Supplemental Fig. 3b.

We next evaluated the specific cytotoxicity of SYK-DC–CTLs against two groups of cells, using WT-DC–CTLs and GFP-DC–CTLs as controls. Group one included SYK-negative cell lines (MDA-MB-231, MCF-10A, and hTERT-RPE1), while group two included SYK-positive cell lines (MCF-7 and RB-Y79). Our flow cytometry results showed that the group two SYK-positive cell lines showed higher levels of cytotoxicity upon co-culture with SYK-DC–CTLs compared with the control WT-DC–CTLs and GFP-DC–CTLs. In contrast, the group one SYK-negative cell lines showed no significant differences in cytotoxicity upon co-culture with SYK-DC–CTLs or the controls (Fig. 2e). The flow cytometry scatter plots of the cytotoxicity experiments are shown in Supplemental Fig. 4.

Next, we analyzed the cytotoxic activity of SYK-DC–CTLs against GFP stably expressing RB-Y79 cells (RB-Y79-GFP) (Liu et al. 2013) by video microscopy. Figure 2f shows a RB-Y79-GFP cell attacked by several SYK-DC–CTL cells. The disappearance of GFP fluorescence occurred about 1 h after co-culture, which means that the target cells were induced to undergo apoptosis by SYK-DC–CTLs. Quantification of the live imaging results is shown in Fig. 2g. After 50 min of SYK-DC–CTLs and RB–Y79–GFP co-culture, the mean gray value of fluorescence in target cells GFP-K562 slumped. Furthermore, compared to the method using tumor lysis antigen-loaded DC–CTLs, we measured the efficiency of SYK-DC–CTL that resulted in a 50% increasing of cytotoxicity against RB-Y79 cells (Supplemental Fig. 5a, c) as well as a twice ratio of RB-Y79 cells apoptosis (Supplemental Fig. 5b, d).

### Expression of SYK in RB-Y79 cells in vitro mediates SYK-DC cytotoxicity

**Fig. 3:**
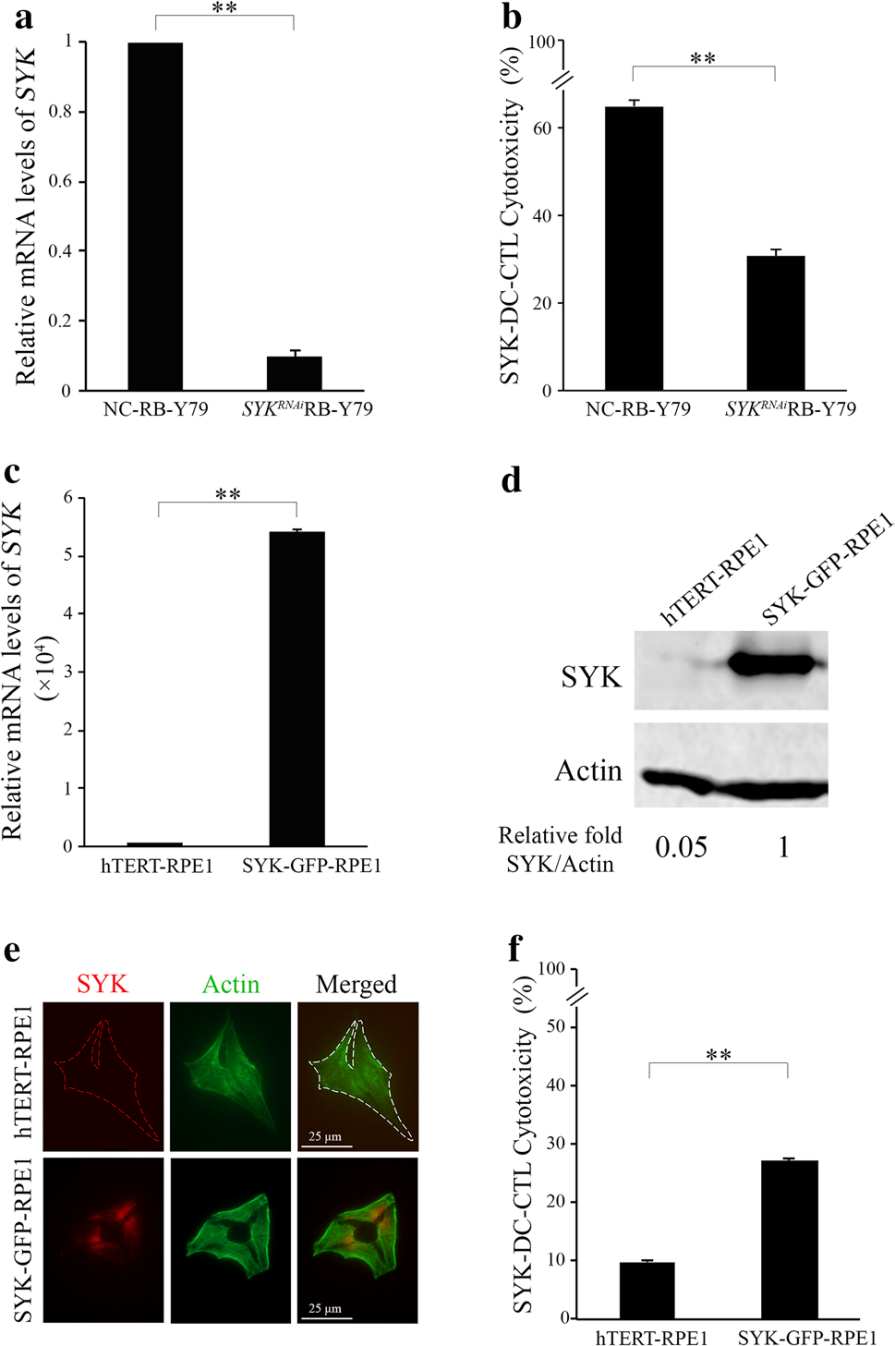
Expression of SYK on RB-Y79 cells in vitro mediates T-lymphocyte-specific cytotoxicity. **a** Quantification of qRT-PCR analysis of *SYK* in RB-Y79 cells transfected with siRNA against SYK (*SYK*^RNAi^RB-Y79 cells) or control siRNA (NC-RB-Y79 cells). *GAPDH* was used as the internal control gene. **b** Flow cytometry analysis of the cytotoxic activity of SYK-DC–CTLs against NC-RB-Y79 and *SYK*^RNAi^RB-Y79 cells. Data were mean ± SEM from three independent experiments. **p < 0.01. **c** Quantification of qRT-PCR analysis of *SYK* in SYK–GPF–RPE1 cells and wild-type hTERT-RPE1 cells. *GAPDH* was used as the internal control. **d** The relative SYK protein expression levels in hTERT-RPE1 and SYK–GFP–RPE1 cells were determined by western blotting. Actin was used as a loading control. **e** Immunofluorescent staining of monoclonal SYK antibody showed that SYK (red signal) existed on the surface of SYK–GFP–RPE1 cells (bottom panel) but not on that of hTERT-RPE1 cells (top panel). Scale bar equals 25 µm. **f** Flow cytometry analysis of the cytotoxic activity of SYK-DC–CTL cells against hTERT-RPE1 and SYK–GFP–RPE1 cells. Data were mean ± SEM from three independent experiments. **P < 0.01

To further characterize the specific cytotoxicity of SYK-DC–CTLs against RB-Y79 cells, we silenced *SYK* in RB-Y79 cells (*SYK*^RNAi^RB-Y79) and confirmed significantly lower *SYK* levels in *SYK*^RNAi^RB-Y79 cells than negative control siRNA-treated RB-Y79 (NC-RB-Y79) cells (*p* < 0.01) (Fig. 3a). We observed differences in viability when using siRNA for the silence of *SYK* in the RB-Y79 cell line (data shown in Supplemental Fig. 5e). NC-RB-Y79 was observed spontaneous mortality of 1.35%, and *SYK*^RNAi^RB-Y79 cell group presented mortality of 4.56%. However, the mortality of SYK-DC–CTL treated *SYK*^RNAi^RB-Y79 cell group is more than 30%, which means the cell death resulted from the specific cytotoxicity of T cells. To calculate the cytotoxicity in our experiments, we have already deduced the spontaneous mortality of NC–RB-Y79 and *SYK*^RNAi^RB-Y79 cells in Fig. 3b. SYK-DC–CTLs had remarkably less cytotoxic activity against *SYK*^RNAi^RB-Y79 cells compared with the NC-RB-Y79 cells. To further characterize the key role of SYK in the specific cytotoxicity of SYK-DC–CTLs, we overexpressed SYK in SYK-negative hTERT-RPE1 cells and confirmed increasing of relative mRNA levels of *SYK* (Fig. 3c) and elevated SYK expression (Fig. 3d) in SYK–GFP–RPE1 cells compared with wild-type hTERT-RPE1 cells. Moreover, the SYK protein was confirmed to be existed on the surface of SYK–GFP–RPE1 cells (Fig. 3e) using SYK monoclonal antibody rather existing on the surface of wild-type hTERT-RPE1 cells. The cytotoxicity of SYK-DC–CTLs against SYK overexpressed hTERT-RPE1 cells was significantly increased compared with that in wild-type hTERT-RPE1 cells (Fig. 3f). Bundling all above results, it clearly demonstrates that SYK protein expression in RB-Y79 cells can induce specific cytotoxicity by SYK-DC–CTLs.

### SYK-DC–CTLs exhibit specific cytotoxicity against carboplatin-resistant RB-Y79 cells in vitro

Carboplatin is a commonly used chemotherapeutic drug for RB treatment. Although the treatment can be relatively effective at the beginning of the therapy, as the treatment continues, the patients can develop resistance to carboplatin, increasing the chances of tumor regrowth and formation of secondary metastases (Assayag et al. 2016).

**Fig. 4:**
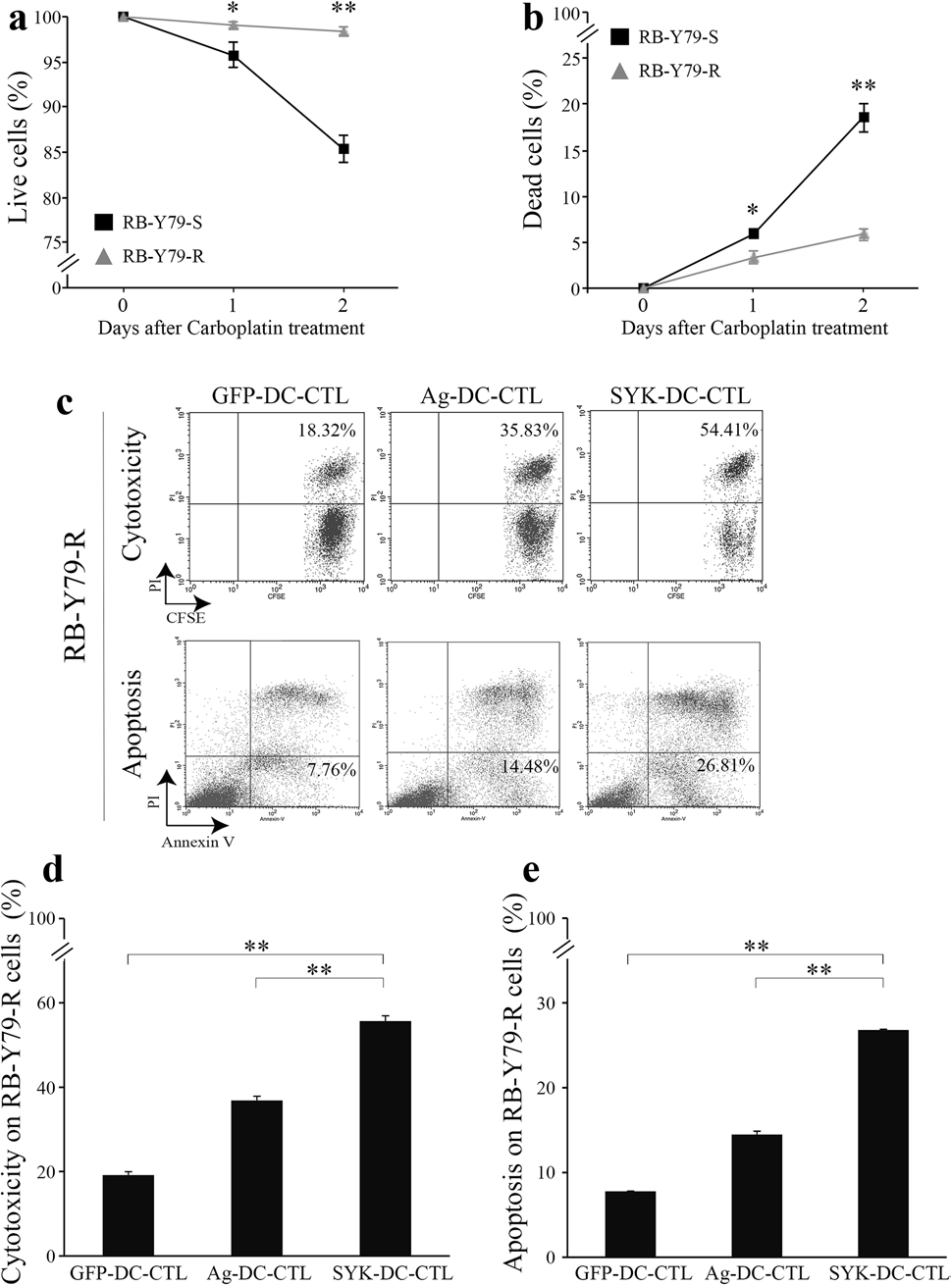
SYK-DC–CTLs exhibit specific cytotoxicity against carboplatin-resistant RB-Y79 cells in vitro. The cell viability of RB-79-S and RB-Y79-R cells in media containing carboplatin was measured by trypan blue staining (**a**) and PI staining (**b**). **c** Cytotoxicity and apoptosis analysis of GFP-DC–CTL cells, Ag-DC–CTL cells and SYK-DC–CTL cells against RB-Y79-R cells were detected by flow cytometry. The figures showed the proportion of dead RB-Y79-R cells (top panel) and apoptotic RB-Y79-R cells (bottom panel). **d** Cytotoxicity of GFP-DC–CTL cells, Ag-DC–CTL cells and SYK-DC–CTL cells against RB-Y79-R cells. **e** Quantification of apoptotic RB-Y79-R cells after co-culture with GFP-DC–CTL cells, Ag-DC–CTL cells or SYK-DC–CTL cells. Data were given as mean ± SEM from three independent experiments. *p < 0.05, **p < 0.01

To assess whether our experimental immunotherapy could be effective against carboplatin-resistant RB cells, we analyzed the cytotoxic activity of SYK-DC–CTLs against carboplatin-resistant RB-Y79 cells (RB-Y79-R) (Wang et al. 2013). We first confirmed resistance to carboplatin by culturing RB-Y79-sensitive (RB-79-S) and RB-Y79-Resistant cells(RB-Y79-R) with 40 µg/ml carboplatin for 24 and 48 h and examining cell viability using trypan blue staining (Fig. 4a) and PI staining (Fig. 4b). RB-Y79-R cells largely survived in the presence of carboplatin (~ 98% survival rate after 48 h) while 80% of RB-Y79-S survived during the same time interval (Fig. 4a, b).

We next evaluated the ability of SYK-DC–CTLs to induce cytotoxicity of RB-Y79-R cells. Ag-DC–CTLs were used as control to judge the intensity of SYK-DC–CTL’s cytotoxicity. Our results revealed that the cytotoxicity of GFP-DC–CTLs, Ag-DC–CTLs, and SYK-DC–CTLs against RB-Y79-R cells was 19.14 ± 0.82, 36.84 ± 1.01, and 55.67 ± 1.26%, respectively (Fig. 4c–e). Compared with Ag-DC–CTLs, SYK-DC–CTL showed a stronger cytotoxic effect against RB-Y79-R cells.

## Discussion

The major limitations to achieving effective cancer treatments are the lack of specificity in targeting cancer cells and reducing side effects. Thus, the identification of specific targets has become a necessity for the development of effective targeted therapies. Based on the recent report identifying *SYK* as an important oncogene in RB (Zhang et al. 2012), we evaluated the use of SYK protein as a putative target to deliver selective immunotherapy against RB cells. Importantly, this study is the first report on the expression of SYK on DCs and the first demonstration that SYK expression confers specificity of immunoreactivity against RB cells with no immunoreactivity on normal retina cells in vitro.

In our study, the HLA histocompatibility seems important for the DC–CTL response. However, we noticed that the MCF-7 cell line using as SYK-positive control was recognized by the T cells although this cell line is not HLA-A2 positive. The reasonable explanation could be that HLA-A2 is one of HLA class I family which consists of HLA-A, HLA-B, and HLA-C (Rabasa et al. 2017). Although the MCF-7 cell line is not HLA-A2 positive, SYK antigen could be presented by the other HLA subtypes.

In agreement with the previous study (Zhang et al. 2012), our data confirm that the expression level of *SYK* is low in normal retina cells but is high in an RB cell line (RB-Y79). Although previous works showed that SYK accumulated in the cytoplasm in RB cells (Zhang et al. 2012), our immunofluorescent staining results showed SYK expression on the cell surface. SYK protein localization on the cell membrane facilitates activated CTLs to specifically recognize the antigens present on the surface of RB-Y79 cells. This is key for specific lymphocyte-based immunotherapy. SYK is also a member of a family of cytoplasmic non-receptor tyrosine kinases. This confers an additional advantage for its use in targeted cell immunotherapy because RB-Y79 cells express the SYK protein on the cell surface, allowing it to be recognized by cytotoxic immune cells, while hematopoietic cells, which express cytoplasmic SYK, may be protected from SYK-driven immune cytotoxic activity (Chauhan et al. 2016). Although we demonstrated *SYK* gene expression in SYK-DCs by qRT-PCR, we failed to detect SYK protein on the surface of infected DC cells using flow cytometry (data not shown). The possible reasons why we failed to detect the SYK signal in DCs could due to two aspects. Firstly, in a classical pathway in DCs, the antigen is cleaved into peptides by endosomal proteases, particularly cathepsin S, and bound by class I molecules probably in the endocytic compartment itself (Rock and Shen 2005). Secondly, the processed antigen could not be presented everlastingly on the DC surface, published data have also explained how duration of antigen presentation affects the dynamics of T-cell–DC interactions and consequently determine immune response (Benson et al. 2015). Thus, it is easy to understand why we see no staining for SYK on the pulsed DCs.

Cell immunotherapy has advantages over the use of drugs for cancer treatment, as immunotherapy is highly tumor-specific and, for the most part, lacks undesirable side effects. Our results also suggest that SYK-DC–CTLs may also be used to treat other tumors that express SYK protein on the surface of cancerous cells. SYK is upregulated in human prostate cancer and is associated with malignant progression (Ghotra et al. 2015). SYK methylation was also correlated with poor overall survival of colorectal cancer (Yang et al. 2013) and SYK is also overexpressed in ovarian cancer (Sultan et al. 2011). Further studies will be needed to shed light on the use of SYK for targeted cancer therapy.

Based on our findings, we propose that designed lentivirus-targeted immunotherapy may provide a foundation for therapeutic innovation against a broad spectrum of cancers, especially leukemia, lymphomas (Ma et al. 2015) and RB treatment (this paper). However, the exact curative effect of SYK-DC–CTL immunotherapy still needs to be demonstrated through animal experiments and clinical trials.

In summary, we found that *SYK*-modified DC–CTLs were specific and highly cytotoxic against multiple SYK-positive cell lines, especially RB cells, while they had a minimal effect on SYK-negative cell lines including normal retinal cells. This suggests a promising specific and safe approach for RB treatment. Our study constitutes one of the first demonstrations that lentivirus-targeted immunotherapy may be an effective means for treating cancers and indicates that SYK may prove to be a suitable candidate for targeted therapy of RB.

## Conclusions

In conclusion, our data suggest that SYK could be a potential immunotherapy target antigen presented by DCs. These observations indicate that CTLs were largely activated by SYK overexpressed DCs with specific cytotoxicity on RB-Y79 cells. Moreover, we propose SYK as a candidate target for treatment of chemoresistant RB.

## Electronic supplementary material

Below is the link to the electronic supplementary material.

**Supplemental Fig. 1** (a) The *GFP* and *SYK* gene expression real-time amplification curves of WT-DC, GFP-DC and SYK-DC cells. *GAPDH* was used as the internal control gene. (b) The *SYK* gene expression real-time amplification curves of hTERT-RPE1 and RB-Y79 cells. *18S rRNA* was used as the internal control gene.

**Supplemental Fig. 2** The flow cytometry scatter plots showed the phenotypes of WT-DC–CTL, GFP-DC–CTL, and SYK-DC–CTL cells. (TIF 7937 KB)

**Supplemental Fig. 3** (a) Using only secondary antibody goat anti-rabbit conjugated with fluorescein isothiocyanate to stain RB-Y79 cells, the left panel showed no green signals were detected. (b) The flow cytometry scatter plots showed the proportions of PBMC, MDA-MB-231, MCF-10A, MCF-7, hTERT-RPE1, and RB-Y79 cells that express HLA-A2. (TIF 3278 KB)

**Supplemental Fig. 4** The flow cytometry scatter plots showed the cytotoxicity of WT-DC–CTL, GFP-DC–CTL and SYK-DC–CTL cells against MDA-MB-231, MCF-10A, MCF-7, hTERT-RPE1, and RB-Y79 cells. (TIF 7902 KB)

**Supplemental Fig. 5** (a-d) The column graphs showed the cytotoxicity (a) and apoptosis (b) of Ag-DC–CTL and SYK-DC–CTL cells against hTERT-RPE1 and RB-Y79 cells, respectively. The flow cytometry scatter plots showed the cytotoxicity (c) and apoptosis (d) of Ag-DC–CTL and SYK-DC–CTL cells against RB-Y79 cells (top panel) and hTERT-RPE1 cells (bottom panel), respectively. (e) Flow cytometry scatter plots showed the spontaneous mortality of RB-Y79 and *SYK*^RNAi^RB-Y79 cells. (TIF 35891 KB)
