# Supplementary figures and images for: SYK-targeted dendritic cell-mediated cytotoxic T lymphocytes enhance the effect of immunotherapy on retinoblastoma
Source: J Cancer Res Clin Oncol. 2018 Jan 25;144(4):675–84. doi: 10.1007/s00432-018-2584-x (PMC5843685; doi:10.1007/s00432-018-2584-x)

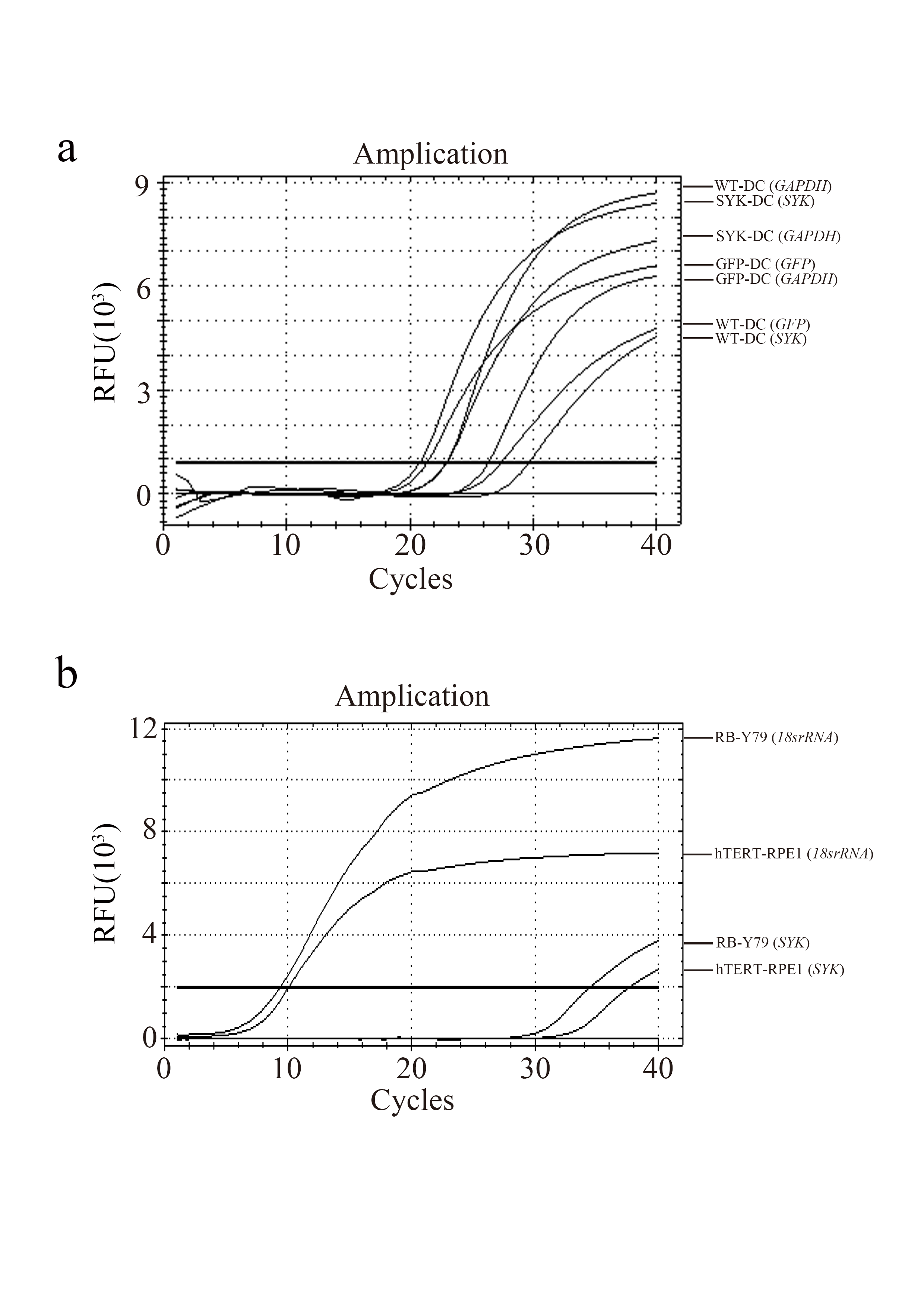

Supplement: Supplementary file 1 — Supplemental Fig. 1 (a) The GFP and SYK gene expression real-time amplification curves of WT-DC, GFP-DC and SYK-DC cells. GAPDH was used as the internal control gene. (b) The SYK gene expression real-time amplification curves of hTERT-RPE1 and RB-Y79 cells. 18S rRNA was used as the internal control gene. [file 432_2018_2584_MOESM1_ESM.tif]

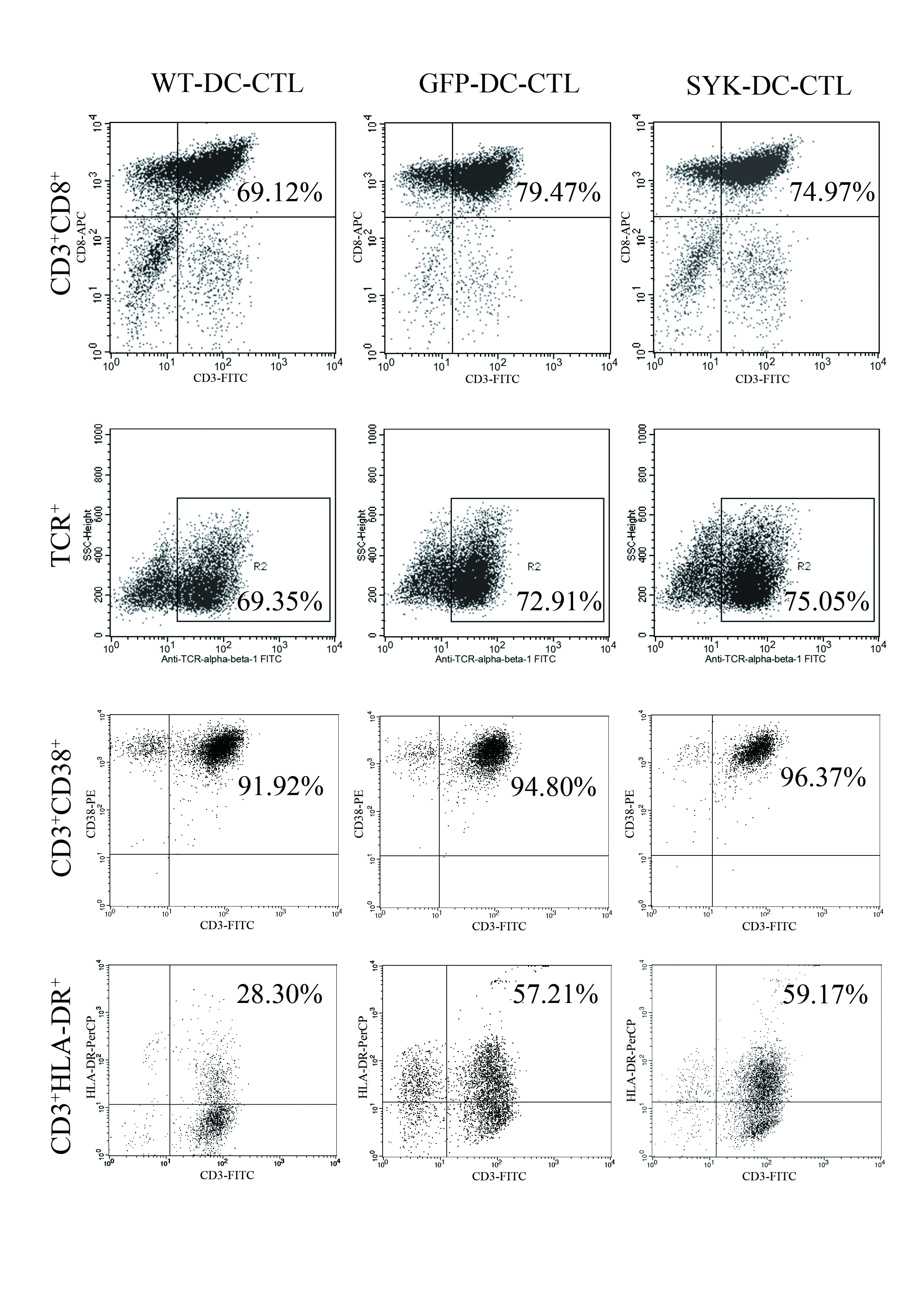

Supplement: Supplementary file 2 — Supplemental Fig. 2 The flow cytometry scatter plots showed the phenotypes of WT-DC–CTL, GFP-DC–CTL, and SYK-DC–CTL cells. (TIF 7937 KB) [file 432_2018_2584_MOESM2_ESM.tif]

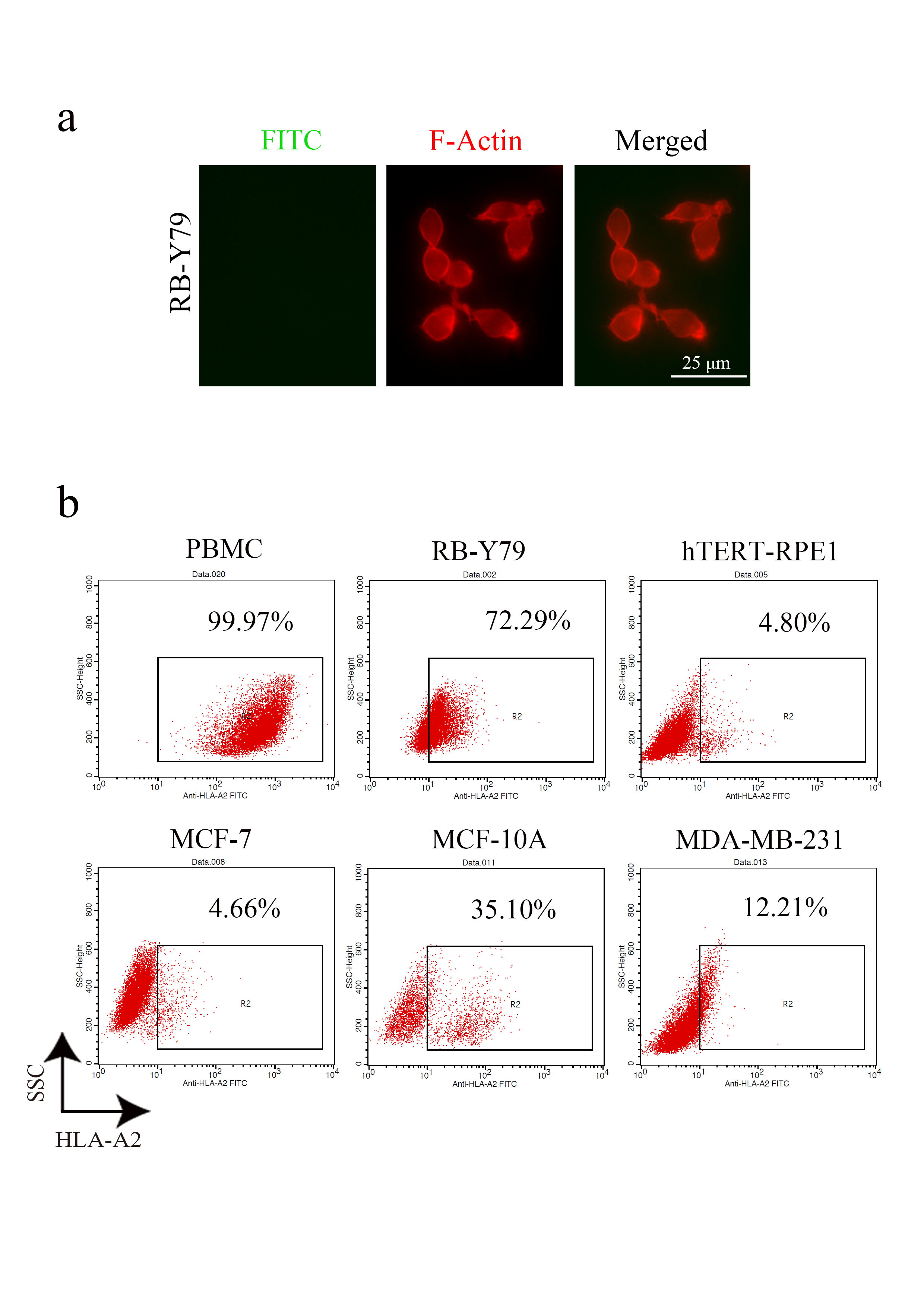

Supplement: Supplementary file 3 — Supplemental Fig. 3 (a) Using only secondary antibody goat anti-rabbit conjugated with fluorescein isothiocyanate to stain RB-Y79 cells, the left panel showed no green signals were detected. (b) The flow cytometry scatter plots showed the proportions of PBMC, MDA-MB-231, MCF-10A, MCF-7, hTERT-RPE1, and RB-Y79 cells that express HLA-A2. (TIF 3278 KB) [file 432_2018_2584_MOESM3_ESM.tif]

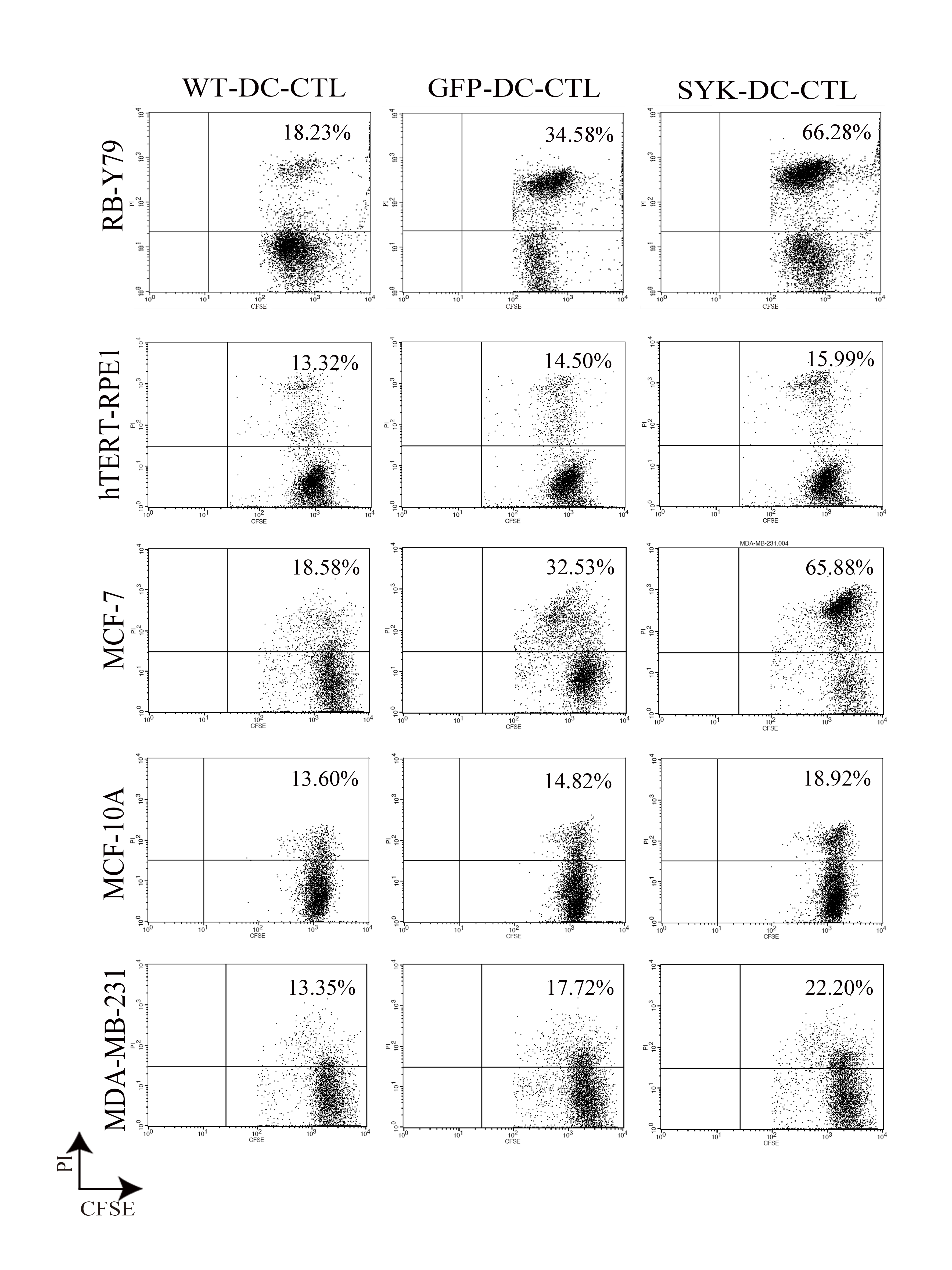

Supplement: Supplementary file 4 — Supplemental Fig. 4 The flow cytometry scatter plots showed the cytotoxicity of WT-DC–CTL, GFP-DC–CTL and SYK-DC–CTL cells against MDA-MB-231, MCF-10A, MCF-7, hTERT-RPE1, and RB-Y79 cells. (TIF 7902 KB) [file 432_2018_2584_MOESM4_ESM.tif]

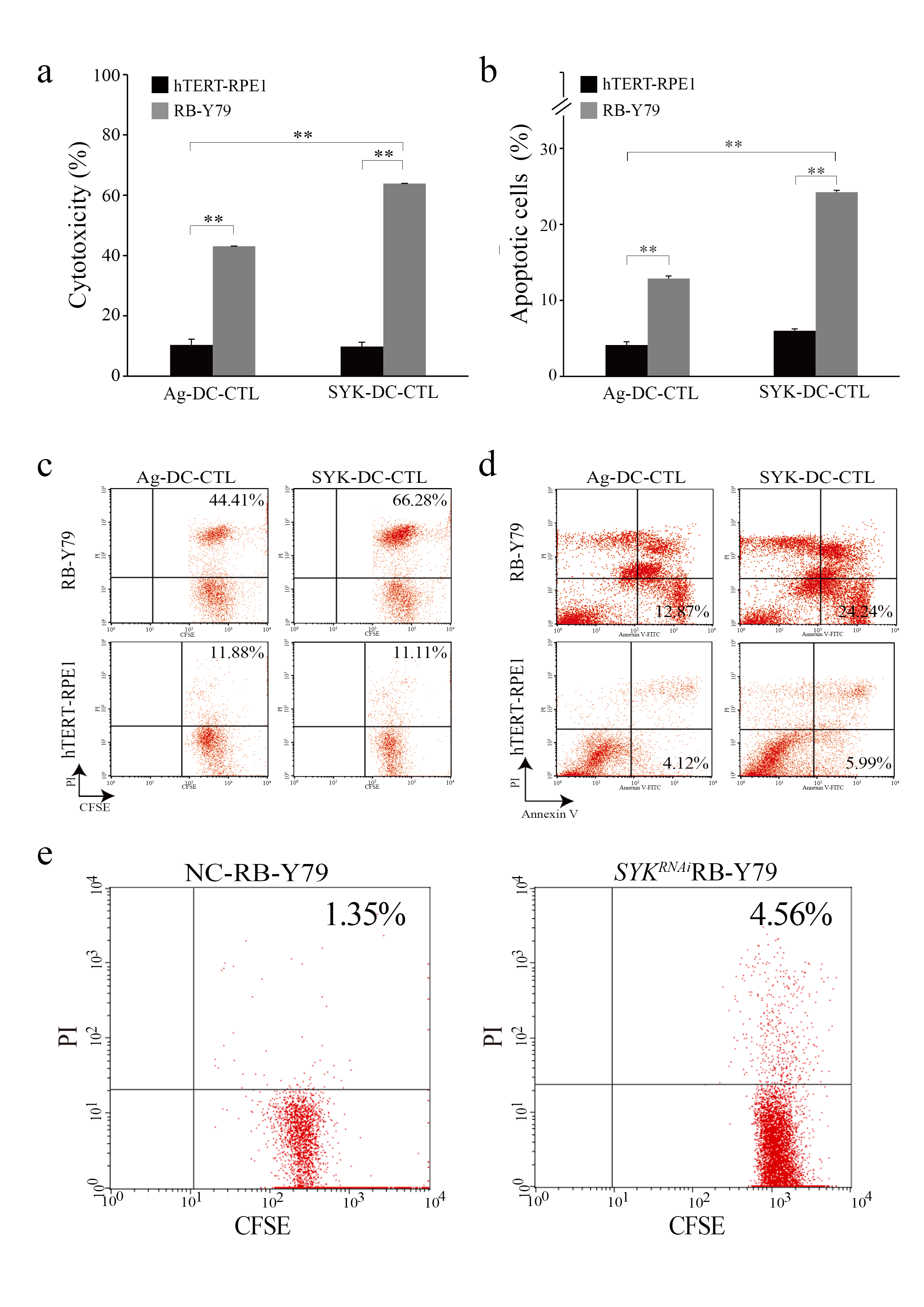

Supplement: Supplementary file 5 — Supplemental Fig. 5 (a-d) The column graphs showed the cytotoxicity (a) and apoptosis (b) of Ag-DC–CTL and SYK-DC–CTL cells against hTERT-RPE1 and RB-Y79 cells, respectively. The flow cytometry scatter plots showed the cytotoxicity (c) and apoptosis (d) of Ag-DC–CTL and SYK-DC–CTL cells against RB-Y79 cells (top panel) and hTERT-RPE1 cells (bottom panel), respectively. (e) Flow cytometry scatter plots showed the spontaneous mortality of RB-Y79 and SYKRNAiRB-Y79 cells. (TIF 35891 KB) [file 432_2018_2584_MOESM5_ESM.tif]
